# A state-of-the-art review on LSD1 and its inhibitors in breast cancer: Molecular mechanisms and therapeutic significance

**DOI:** 10.3389/fphar.2022.989575

**Published:** 2022-09-16

**Authors:** Guan-Jun Yang, Yan-Jun Liu, Li-Jian Ding, Fan Tao, Ming-Hui Zhu, Zhen-Yuan Shi, Juan-Ming Wen, Meng-Yao Niu, Xiang Li, Zhan-Song Xu, Wan-Jia Qin, Chen-Jie Fei, Jiong Chen

**Affiliations:** ^1^ State Key Laboratory for Managing Biotic and Chemical Threats to the Quality and Safety of Agro-products, Ningbo University, Ningbo, Zhejiang, China; ^2^ Laboratory of Biochemistry and Molecular Biology, School of Marine Sciences, Ningbo University, Ningbo, China; ^3^ Key Laboratory of Aquacultural Biotechnology Ministry of Education, Ningbo University, Ningbo, China; ^4^ Li Dak Sum Yip Yio Chin Kenneth Li Marine Biopharmaceutical Research Center, Department of Marine Pharmacy, College of Food and Pharmaceutical Sciences, Ningbo University, Ningbo, China

**Keywords:** LSD1, histone demethylase, breast cancer, inhibitors, H3K4me1/2, H3K9me1/2

## Abstract

Breast cancer (BC) is a kind of malignant cancer in women, and it has become the most diagnosed cancer worldwide since 2020. Histone methylation is a common biological epigenetic modification mediating varieties of physiological and pathological processes. Lysine-specific demethylase 1 (LSD1), a first identified histone demethylase, mediates the removal of methyl groups from histones H3K4me1/2 and H3K9me1/2 and plays a crucial role in varieties of cancer progression. It is also specifically amplified in breast cancer and contributes to BC tumorigenesis and drug resistance *via* both demethylase and non-demethylase manners. This review will provide insight into the overview structure of LSD1, summarize its action mechanisms in BC, describe the therapeutic potential of LSD1 inhibitors in BC, and prospect the current opportunities and challenges of targeting LSD1 for BC therapy.

## 1 Introduction

Breast cancer (BC) is a kind of malignant tumour in women occurring in the breast glandular epithelial tissues, and it has become the most diagnosed cancer worldwide since 2020 (Siegel et al., 2021; Siegel et al., 2022). The incidence of BC is increasing year by year and patients are getting younger and younger, posing a serious threat to women’s health (Siegel et al., 2022). Although advances in early diagnosis and treatment of BC have partially alleviated the crisis of some BC patients, there are still a large number of patients suffering from BC due to their complex pathogenesis, insensitivity to existing drugs, and easy-to-develop drug resistance (Yang et al., 2019; Yang et al., 2021b). Therefore, there is an urgent need to find new and effective targeted therapies for this type of BC.

**FIGURE 1:**
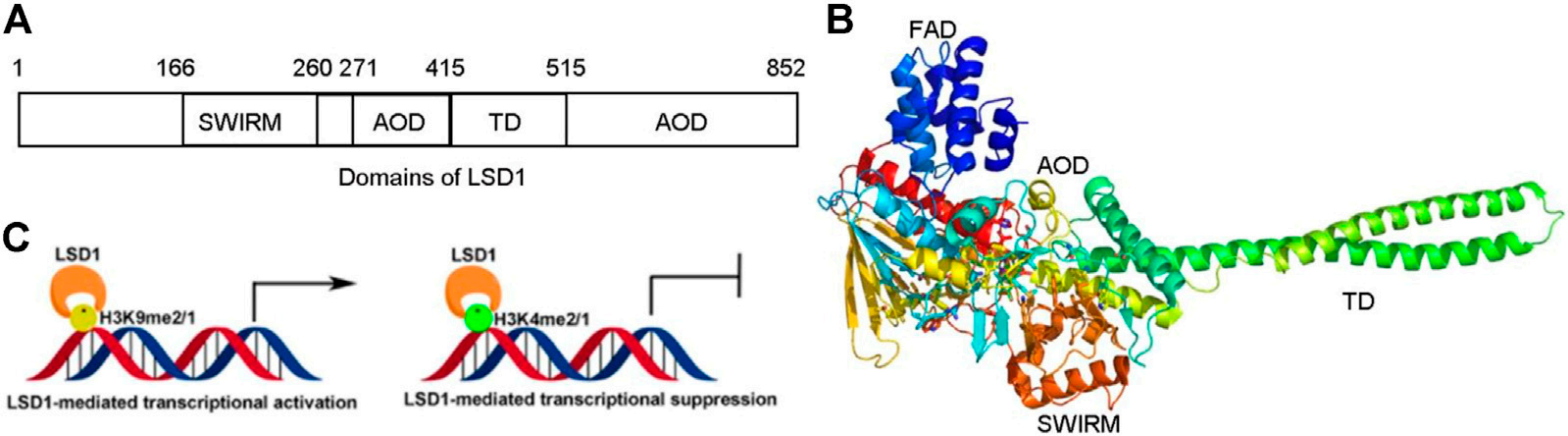
Structure and function of LSD1. **(A)** Distribution of domains of LSD1. **(B)** LSD1-mediated transcriptional modulation. **(C)** Overview structure of LSD1.

Lysine-specific demethylase 1 (LSD1) is a flavin-dependent lysine-specific histone demethylase first identified in 2004 and mediated transcriptional activation or repression *via* erasing methyl groups from H3K9me2/1 and H3K4me2/1, respectively (Figure 1) (Shi et al., 2004; Fang et al., 2020; Kim et al., 2021; Malagraba et al., 2022; Song et al., 2022). Recently studies found that LSD1 could also remove the methyl groups from several non-histone proteins such as ERα, MTA1, HIF-1α, AGO2, HSP90, MEFD2, and STAT3 and be involved in many cancer cell events (Majello et al., 2019). LSD1 exhibits its catalytic mechanism *via* consuming oxidation of FAD and O_2_ and yielding HCHO and H_2_O_2_ *in cellulo* (Yang et al., 2018a). Apart from demethylase activity, LSD1 exhibits non-demethylase activity *via* interacting with its client proteins and is involved in physiological and pathological processes (Gu et al., 2020). LSD1 is also overexpressed in varieties of cancers and mediates their progression (Yang et al., 2018a; Fang et al., 2019; Fang et al., 2020). LSD1 is aberrantly expressed in BC and promotes proliferation and metastasis of BC cells (Liu et al., 2020a; Zhou et al., 2021a). Moreover, LSD1 also is involved in regulating resistance of chemotherapy and immunotherapy in BC (Kim et al., 2013; Boulding et al., 2018; Yang et al., 2018d; Qin et al., 2019; Verigos et al., 2019; Tu et al., 2020; Sobczak et al., 2022). Given the multifaceted functions of LSD1 in BC progression, new therapeutic strategies targeting LSD1 are constantly being developed, such as the discovery of novel LSD1 inhibitors, the development of dual-target inhibitors, and the combination therapies with chemical agents or immunomodulators (Kim et al., 2013; Boulding et al., 2018; Yang et al., 2018d; Qin et al., 2019; Verigos et al., 2019; Tu et al., 2020; Sobczak et al., 2022). Therefore, LSD1 is a potential target for BC therapy.

Herein, the structures, functions and the regulatory roles of LSD1 in tumorigenesis were introduced, the reported LSD1 inhibitors and their therapeutic mechanisms for BC treatment were summarized, and the current challenges and the potential opportunities of LSD1 inhibitors for BC therapy were prospected.

## 2 The overview of LSD1

### 2.1 The structure of LSD1

LSD1 is a FAD-dependent demethylase encoding a peptide with 852 amino acid residues. LSD1 consists of highly conserved three distinct domains: a SWI3/Rac8/Moira (SWIRM) domain, a tower domain (TD), and a catalytic amine oxidase domain (AOD) (Figures 1A,B) (Forneris et al., 2008; Yang et al., 2018a). The SWIRM domain is an indispensable domain for LSD1-mediated histone modification and chromatin remodeling (Metzger et al., 2005). The TD is a special domain with two antiparallel helices, and it can bind to RCOR1 and form the CoREST complex (Pilotto et al., 2015; Marabelli et al., 2016). The AOD domain is the catalytic domain of LSD1, and it consists of two well-defined motifs: the substrate-recognition motif and the FAD-binding motif (Yang et al., 2018a). The latter is highly conserved and responsible for binding sites of some reported LSD1 inhibitors. The two motifs assemble into a big cavity containing the interface of enzyme activity centre (Forneris et al., 2008). In the active state of LSD1, the second lobe of the AOD domain could form a hydrophobic binding pocket SWIRM domain (Yoneyama et al., 2007). This binding pocket mediates LSD1 binding to histone H3 and is also chosen as the binding pocket for developing LSD1 inhibitors (Zhou et al., 2015).

### 2.2 The functions and regulations of LSD1

LSD1 plays heterogeneous roles *via* transcriptionally modulating its downstream genes in demethylase-dependent or -independent modes in varieties of cancers (Song et al., 2022; Yang et al., 2022). It acts as an oncogene in some cancers, while functioning as a cancer-suppressor gene in the other cancers (Yang et al., 2018a; Hu et al., 2019). In addition, LSD1 is also regulated by multiple epigenetic regulators in BC.

#### 2.2.1 LSD1 as a transcription co-repressor

Upregulating of H3K4me2/1 often contributes to the transcriptional activation (Figure 1C). LSD1 removes methyl groups from active Histone 3 *via* assembling into different co-repressor complexes with several distinct proteins and shaping chromatin into a repressive conformation. For example, LSD1 was found to form a co-repressor complex with SIN3A/HDAC and maintain sensitivity to chemotherapy *via* reducing inhibiting several genes such as *TGFB2*, CASP7, *TERT*, *MDM2*, and *HIF1α* in BC (Yang et al., 2018d). It also could assemble into LSD1/CoREST/BRMS1 and inhibit the metastasis of BC cells *via* reduced levels of *Vimentin*, *COL5A2*, *INSIG2*, *MRPL33*, *SLC1A1, KLK11*, and *OLFML3* (Qiu et al., 2018).

#### 2.2.2 LSD1 as a transcription co-activator

LSD1 also works as a transcriptional co-activator *via* demethylating H3K9me2/1 (Figure 1C). It can promote estrogen transcription in breast cancer cells through interacting with estrogen receptor (ER) (Bennesch et al., 2016). Additionally, LSD1 also regulates chromatin events such as DNA replication, heterochromatin, and imprinting propagation (Zhu et al., 2012; Yang et al., 2018a).

#### 2.2.3 The epigenetic regulation of LSD1

**FIGURE 2:**
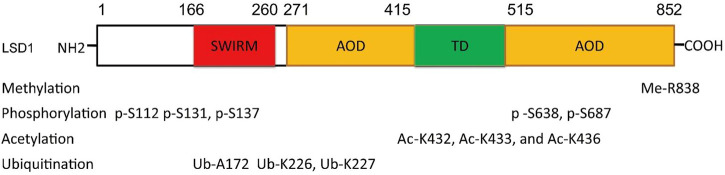
Residue sites were modified by varieties of posttranslational modifications in LSD1.

The function of LSD1 has also been found to be regulated by many epigenetic components. For example, miR-708 inhibits BC proliferation and invasion *via* directly binding to the 3′ UTR of LSD1 and reducing its level (Ma et al., 2016). In addition, epigenetic modifications such as phosphorylation (Peng et al., 2015; Feng et al., 2016; Zhou et al., 2016), acetylation (Luo et al., 2016), methylation (Liu et al., 2020a), and ubiquitination (Wu et al., 2013; Yi et al., 2016; Zhou et al., 2016; Gong et al., 2021) also contribute to the function of LSD1 (Figure 2). PKCα can phosphorylate LSD1 at S112, activating its demethylase activity and enhancing the occupancy on *E-cadherin* promoter, and finally promote EMT and metastasis in BC (Feng et al., 2016). Phosphorylated modification is also found to regulate LSD1-medatied DNA damage (Peng et al., 2015). To be specific, LSD1 was di-phosphorylated at S131 and S137 by CK2 and Wip1, respectively, which promoted its interaction with RNF168 and RNF168-dependent 53BP1 ubiquitination and subsequent recruitment to the DNA damage sites. CK1α was also found to phosphorylate LSD1 at S687 and then induce S683 phosphorylation of LSD1 by nuclear GSK3β (Zhou et al., 2016). The di-phosphorylated LSD1 would be deubiquitylated by USP22 and induced stabilization itself. MOF, a lysine acetyltransferase first found acetylated histone H4 at K16 residue, also could catalyze the acetylation of LSD1 at K432, K433, and K436 and suppress LSD1-medaited EMT in epithelial cells (Luo et al., 2016). Arginine methyltransferase 4 (PRMT4) was also found to mediate deubiquitination and stabilization of LSD1 *via* dimethylating it at R838 and promoting its deubiquitination by USP7 (Liu et al., 2020a). Apart from USP7 and USP22, USP28 also could remove ubiquitin modifications from LSD1, maintain its stability, and thus confer stemness to BC cells (Wu et al., 2013). Recently, OTUD7B was also found to mediate deubiquitination of LSD1 at K226/277 residues, which stabilized LSD1 and promoted its assembly into co-repressor complex (Gong et al., 2021).

## 3 The role of LSD1 in breast cancer

### 3.1 Role of LSD1 in breast cancer progression

**FIGURE 3:**
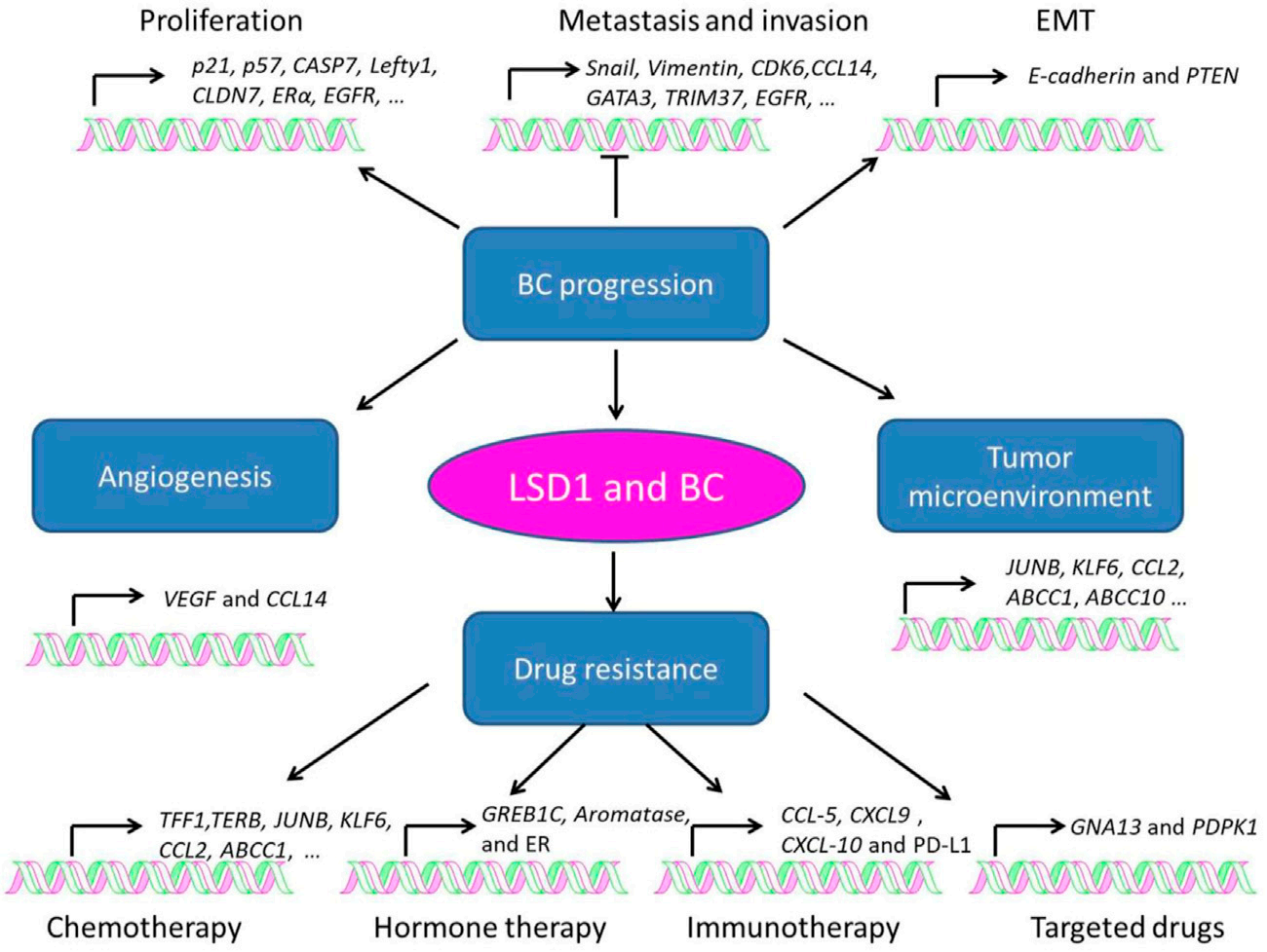
Role of LSD1 in breast cancer progression, angiogenesis, tumor microenvironment, and drug resistance.

LSD1 is overexpressed in several subtypes of BC and functions as an oncogene mediating proliferation, differentiation, invasion, and metastasis of BC cells (Figure 3; Tables 1–3) (Ma et al., 2016; Feng et al., 2017; Yang et al., 2018a; Hu et al., 2019; Zhou et al., 2021a; Ji et al., 2021). When normal human mammary epithelial cells are exposed to carcinogens, their LSD1 levels would be upregulated and promote the carcinogenesis *via* reducing p57^kip2^ level (Bradley et al., 2007). LSD1 exhibits its oncogene functions *via* interacting with distinct ligands in different BC subtypes. As a major biomarker of ER-positive BC, ERα and its transcriptional activity are regulated by LSD1 *via* assembling into complex with different ligands to mediate BC proliferation (Lim et al., 2010; Pollock et al., 2012; Zhu et al., 2012; Andresen et al., 2017). For example, CAC1 antagonized LSD1-mediated ERα activation and suppressed the proliferation of BC cells (Kim et al., 2013), while ASXL2 promoted proliferation of BC cells *via* forming a complex ASXL2/LSD1/UTX/MLL to activate ERα activity (Park et al., 2016). In addition, LSD1 reduces tumor suppressor gene *Lefty1 via* interacting with β-catenin in BC cells (Yakulov et al., 2013), and suppresses BC cell growth through binding to histone deacetylases (HDACs) (Huang et al., 2012; Vasilatos et al., 2013). It also sensitizes BC cells to chemotherapy *via* assembling into a complex with SIN3A/HDAC and inhibits BC proliferation and metastasis *via* interacting with HDAC5 (Cao et al., 2017; Cao et al., 2018; Yang et al., 2018d). Further studies found that LSD1 promotes BC metastasis *via* H3K4me2 demethylase occupying the gene promoters of *Snail* and *Slug* and reducing their levels (Lin et al., 2010; Wu et al., 2012; Lin et al., 2014; Phillips and Kuperwasser, 2014; Bai et al., 2017). Interestingly, androgen receptor (AR) is also involved in BC metastasis *via* interacting with LSD1 to reduce *E-cadherin* and upregulate *Vimentin* (Feng et al., 2017).

**TABLE 1:** Roles of LSD1 in BC progression.

Substrates	Complexes/pathways	Target genes	Functions	References
H3K4me2/me1	—	*p57kip2*	Promoting BC initiation	Bradley et al. (2007)
H3K4me2/me1	CAC1/LSD1/ERα	*ERα-target genes*	Inhibiting proliferation	Kim et al. (2013)
H3K9me2/me1	ASXL2/LSD1/UTX/MLL2	*ERα*	Promoting proliferation	Park et al. (2016)
H3K4me2/1	LSD1/β-catenin	*Lefty1*	Promoting proliferation	Yakulov et al. (2013)
H3K4me2/1	LSD1/SIN3A/HDAC	*CASP7, TGFB2, CDKN1A(p21), HIF1A, TERT,* and *MDM*	Sensitizing BC cells to chemotherapy	Yang et al. (2018d)
H3K4me1/me2	LSD1/HDAC5	*p21* and *CLDN7*	Hindering BC proliferation and invasion	Cao et al. (2017), Cao et al. (2018)
H3K4me2	Snail	*E-cadherin, PTEN*	Promoting EMT	Lin et al., 2010, Lin et al. (2014)
H3K4me2	Slug	*BRCA1, ESR1*	Inhibiting invasion	Bai et al., (2017), Phillips and Kuperwasser, (2014)
H3K9me2, H3K4me2	AR	*E-cadherin* and *Vimentin*	Promoting progression and metastasis	Feng et al. (2017)
H3K4me2	LSD1/CoREST	*IGF1R, RHOA,* and *TGFB1*	Inhibiting metastasis	Wang et al. (2009)
H3K4me2	ZNF516/CtBP/LSD1/CoREST	*EGFR*	Inhibiting proliferation and metastasis	Li et al. (2017)
H3K4me1/me2	BRMS1/LSD1/CoREST	*Vimentin*	Suppressing metastasis and invasion	Qiu et al. (2018)
H3K4me1/me2	LSD1/NuRD/SIX3	*WNT1* and *FOXC2*		Zheng et al. (2018)
H3K4me2, H3K4me3	LSD1/NuRD/KDM5B	*CCL14*		Li et al. (2011)
H3K4me2	LSD1/GATA3	*GATA3*, *TRIM37*		Hu et al. (2019)
H3K4me1/me2, H3K9me2	OTUD7B/LSD1	*Snail, CDK6,* …	Promoting metastasis	Gong et al. (2021)
H3K4me1/me2	PKCα/LSD1	*E-cadherin*	Promoting EMT and metastasis	Feng et al. (2016)
H3K4me1/me2	PRMT4/LSD1/USP7	*E-cadherin* and *Vimentin*	Promoting invasion and metastasis	Liu et al. (2020a)

**TABLE 2:** Regulatory roles of LSD1 in BC angiogenesis and microenvironment.

Substrates	Ligand proteins	Complexes/pathways	Target genes	Functions	References
HIFαK391	NuRD	LSD1/NuRD	*VEGF*	Promoting angiogenesis	Lee et al. (2017)
H3K4me2, H3K4me3	KDM5B and NuRD	KDM5B/LSD1/NuRD	*CCL14*	Suppressing angiogenesis	Li et al. (2011)
H3K4me2/1	PKC-θ	LSD1S111/PKC-θ	*JUNB, KLF10, KLF6,* and *CCL2*	Promoting mesenchymal and stem-like signature, and reducing M1 macrophage	Boulding et al. (2018)
H3K4me2	—	—	*CCL5, CXCL -9, -10,* and *PD-L1*	Reducing CD8^+^ T cell infiltration	Bennani-Baiti, (2012), Shen et al. (2022)
H3K4me2, H3K9me2	CoREST	LSD1/CoREST	Macrophage polarization genes	Inhibiting Mф toward a M1-like phenotype in the TME	Benedetti et al. (2019)

**TABLE 3:** Roles of LSD1 in drug resistance of BC.

Resistant agents		Complexes/pathways	Target genes	Functions	References
Chemotherapy	Paclitaxel	CAC1/LSD1/ER	*TFF1, TERB*	Promoting paclitaxel	Kim et al. (2013)
		LSD1S111/PKC-θ	*JUNB*, *KLF10*, *KLF6*, and *CCL2*	Resistance	Boulding et al. (2018)
	Doxorubicin	—	—	Enhancing BC stemness	Verigos et al. (2019)
	Doxorubicin, cisplatin, daunorubicin, and methotrexate	CBP/LSD1	*ABCC1* and *ABCC10*	Promoting drug efflux	Strachowska et al., (2021); Sobczak et al., (2022)
Hormone therapy	Tamoxifen	LSD1/PELP1/ER	*GREB1C*, *Aromatase*	Promoting BC hormone resistance	Cortez et al. (2012)
		LSD1/ER	*ER*	Activating ER transcriptional activity	Benedetti et al. (2019)
Immunotherapy	PD-1 antibody	—	*CCL5*, *CXCL9*, *CXCL10*, and *PD-L1*	Reducing efficacy of PD-1 antibody	Qin et al. (2019)
		EOMES/LSD1	Mф polarization genes	Reducing immune Cell infiltration and increasing checkpoint markers	Qin et al., (2019); Tu et al., (2020)
Targeted therapy	BRD4 inhibitors	BRD4/LSD1/NuRD	*GNA13* and *PDPK1*	Promoting JQ1 resistance	Liu et al. (2022)

Interestingly, LSD1 also exert its function as a tumor suppressor gene *via* forming different complexes with distinct ligand proteins (Li et al., 2017). LSD1 was also found to inhibit proliferation, invasion, and metastasis *in vitro* and *in vivo via* assembling into LSD1/NuRD complex (Wang et al., 2009; Li et al., 2017). This complex exhibits its heterogeneous tumor suppressor functions dependent of the different subunits in varieties of BC cells (Wang et al., 2009; Li et al., 2017). In MCF-7 cells, zinc-finger protein 516 (ZNF516) inhibited *EGFR* transcription, and thus reduced the proliferation and invasion of BC *in vitro* and *in vivo via* targeting CtBP/LSD1/CoREST complex (Li et al., 2017). Breast carcinoma metastasis suppressor 1 (BRMS1) is another gene coordinating with LSD1/NuRD complex to inhibit the metastasis of MCF7 cells (Qiu et al., 2018). In MDA-MB-231 cells, LSD1/NuRD complex suppressed BC tumorigenesis and metastasis *via* recruiting the homeotic protein SIX3 (Zheng et al., 2018). CC chemokine ligand 14 (CCL14) is a chemokine promoting angiogenesis in viral infection and tumor progression (Mortier et al., 2008; Li et al., 2011). KDM5B reduce *CCL14* transcription to impede metastasis *via* targeting LSD1/NuRD complex (Li et al., 2011). In addition, in luminal BC, LSD1 suppressed invasion, migration, and metastasis BC cells *via* raising GATA3 and repressing TRIM37 (Hu et al., 2019).

Apart from as a subunit of many complexes mediating BC progression, the function of LSD1 was also regulated by several epigenetic enzymes in BC (Feng et al., 2016; Liu et al., 2020a; Gong et al., 2021). Feng et al. (2016) highlighted the PKCα*-*mediated phosphorylation of the S112 residue of LSD1 which was crucial for epithelial-mesenchymal transition (EMT) and metastasis of BC cells. Liu et al. (2020a) found that PRMT4 methylated and stabilized LSD1 *via* promoting it and it binding to deubiquitinase USP7 in BC cells. Recently, Gong et al. (2021) revealed that OTUD7B could remove the Poly-Ub Chains of LSD1 at K226/277 residues, maintain the integrity of LSD1/CoREST/HDACs co-repressor complexes, and inhibit BC metastasis.

### 3.2 Role of LSD1 in tumor angiogenesis

Angiogenesis is a pivotal process for BC growth and metastasis (Ayoub et al., 2022). LSD1 is also involved in this process *via* modulating several pathways (Table 2) (Li et al., 2011; Lee et al., 2017). Li et al. (2011) found that LSD1 worked as a co-repressor and suppressed angiogenesis and metastasis of BC cells *via* assembling into a complex with KDM5B and NuRD and reducing the transcription of CCL4. Hypoxia-inducible factor alpha (HIF1α), a transcription factor promoting breast cancer angiogenesis, is also found to be regulated by LSD1/NuRD complex. To be specific, this complex demethylated HIF1α to stabilize it, and then stabilized HIF1α would upregulate the vascular endothelial growth factor (VEGF) *via* cooperating with CBP and metastasis-associated antigen 1, and induce angiogenesis in BC (Lee et al., 2017).

### 3.3 Role of LSD1 in the breast cancer microenvironment

Tumor microenvironment refers to the internal and external environment of tumor cells where they survive, grow, and metastasize (Xiao and Yu, 2021). Tumor stroma consists of several heterogeneous cells such as cancer-associated fibroblasts (CAFs) and macrophages (Mф), which promote tumorigenesis *via* the secretion of varieties of chemokines, cytokines, and growth factors (Disis, 2010). While LSD1 increases CAF burden and reducing innate M1 Mф infiltration at the primary tumor site in BC (Boulding et al., 2018). In TNBC, LSD1 also mediated CD8^+^ lymphocyte trafficking to the tumor microenvironment and reducing Mф polarization toward M1-like phenotype (Qin et al., 2019; Tan et al., 2019; Shen et al., 2022).

### 3.4 Role of LSD1 in drug resistance of breast cancer

Drug resistance is one of the major causes that leads to distant metastasis, poor prognosis, and death of BC (Wen et al., 2020). LSD1 is widely involved in the resistance to chemotherapy (Kim et al., 2013; Boulding et al., 2018; Verigos et al., 2019; Sobczak et al., 2022), hormone therapy (Bennani-Baiti, 2012; Cortez et al., 2012; Benedetti et al., 2019; Sukocheva et al., 2020), immunotherapy (Qin et al., 2019; Tu et al., 2020), and targeted therapy (Strachowska et al., 2021; Liu et al., 2022) of BC (Table 3). Briefly, LSD1 promotes chemoresistance *via* functioning as a co-activator through interacting with different ligand proteins (Kim et al., 2013; Boulding et al., 2018; Verigos et al., 2019; Strachowska et al., 2021). It mediates resistance to hormone therapy *via* activating ER transcriptional activity (Cortez et al., 2012; Benedetti et al., 2019). In addition, LSD1 is also involved in resistance to PD-1 antibody treatment and BRD4 inhibitors *via* its transcriptional inhibitory activity against multiple oncogenes (Qin et al., 2019; Tu et al., 2020; Liu et al., 2022).

## 4 Targeting LSD1 for breast cancer therapy

Considering the crucial roles of LSD1 in BC progression, it has the potential as a therapeutic target for BC treatment. Currently, tens of LSD1 inhibitors have been documented with anti-BC activity with some of these having entered clinical trials. Herein, they were classified into seven subcategories based on their structural characteristics: PCPA-based LSD1 inhibitors, polyamine analogues, natural products, propargylamine derivatives, benzohydrazide derivatives, phenyl oxazole derivatives, and dual-target inhibitors.

### 4.1 PCPA-based LSD1 inhibitors

**FIGURE 4:**
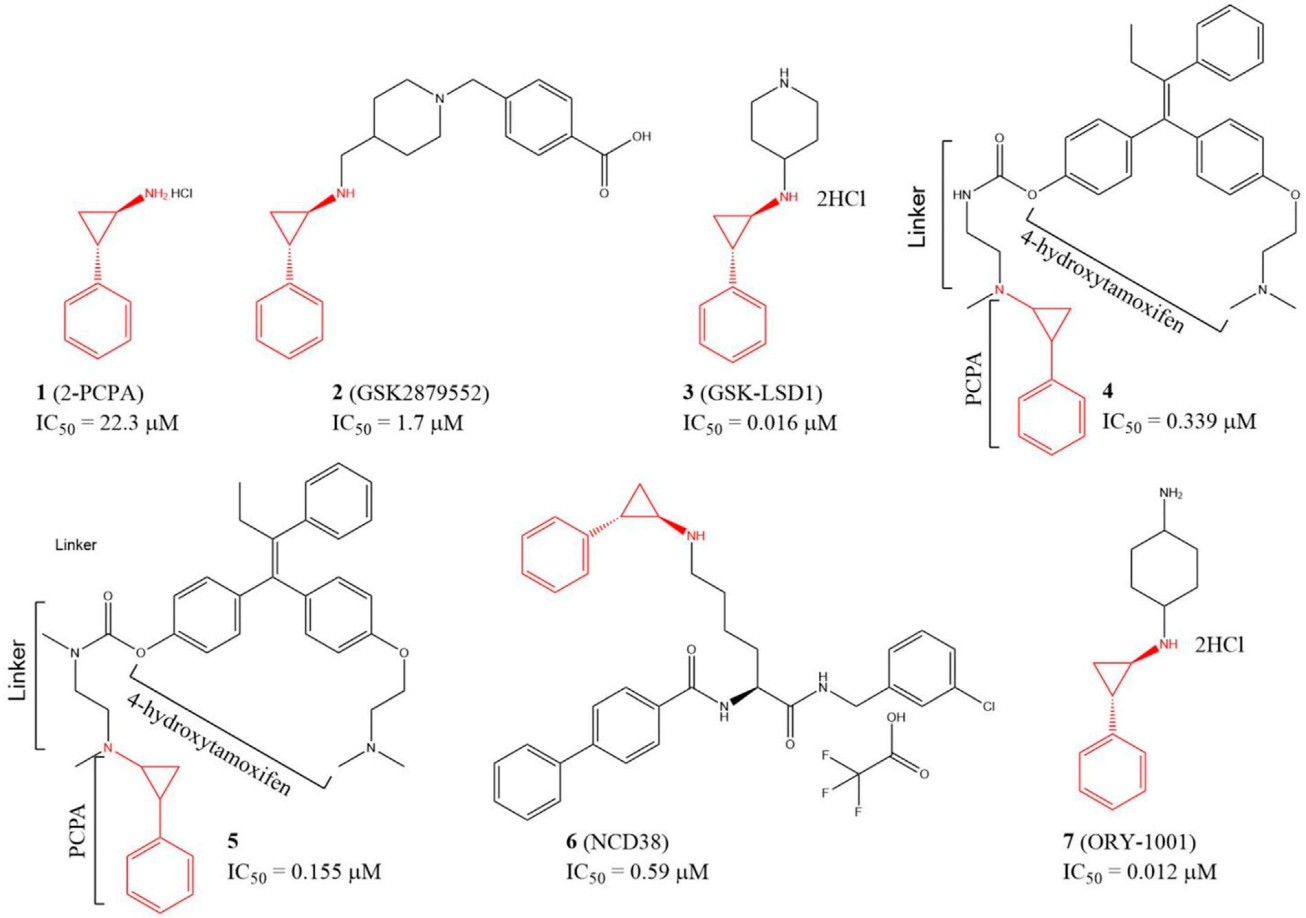
Chemical structures of PCPA-based LSD1 inhibitors in BC therapy.

Since LSD1 and monoamine oxidases (MAOs) share high similarity in their catalytic domains, several LSD1 inhibitors have been discovered based on reported MAO inhibitors *via in silicon* screening and chemical structure optimizations (Yang et al., 2007; Yang et al., 2018a). Tranylcypromine hydrochloride (2-PCPA, **1**), an irreversible monoamine oxidase (MAO) inhibitor with half maximal inhibitory concentration (IC_50_) of 11.5 and 7.0 μM for MAO A and MAO B *in vitro*, respectively, has also exhibited inhibitory activity against LSD1 (IC_50_ = 22.3 μM) (Figure 4) *via* covalently binding to its FAD-binding motif (Ji et al., 2017; Cao et al., 2018). Further study verified that compound **1** also inhibited migration, invasion, and metastasis of TNBC cell lines BT-549 and MDA-MB-231 cells and tumor bone metastasis *in vivo*. In the mechanism, **1** blocked the interaction between LSD1 and slug, and thus upregulated suppressor E-cadherin and reduced epithelial markers (Ferrari-Amorotti et al., 2014)*.* To improve the potency, and selectivity of 2-PCPA, some 2-PCPA derivatives have been designed and synthesized based on multiple optimization strategies. GlaxoSmithKline lnc. has designed 2 PCPA-based LSD1 inhibitors **2** and **3** with IC_50_ of 1.7 μM, and 0.016 μM, respectively, (Figure 4) (Tu et al., 2020; Zhou et al., 2021b). N-alkylated 2-PCPA derivative **2**, a selective and orally bioavailable for LSD1 inhibitor induced IFN-γ/TNF-α-expressing CD8 T cell infiltration into the tumors of 4T1 immunotherapy-resistant mice and sensitized to immunotherapy *via* a LSD1-EOMES switch. Interestingly, 2 showed much potent anti-BC activity than PD-L1 antibody (Tu et al., 2020). Compound **3** promoted the antigen presentation and enhanced the tumor-killing activity of tumor-specific cytotoxic T-cells in 4T1 mouse model (Zhou et al., 2021b). Compounds **4** and **5** (Figure 4) are two PCPA-4-hydroxytamoxifen conjugates, which released 4-hydroxytamoxifen catalyzing by LSD1 *in vitro* and *in cellulo* and exhibited anti-proliferative activity against MCF-7 cells at concentrations as low as 0.1 μM. In addition, both of the two conjugates have better *in cellulo* anti-proliferative activity than their parent compounds (Ota et al., 2016). NCD38 (**6**), a selective LSD1 inactivator optimized from PCPA with IC_50_ of 0.59 μM (Sugino et al., 2017), could reduce the stemness of TNBC cells and tumor growth *in vitro* (Zhou et al., 2021a). ORY-1001 (**7**), a PCPA derivative in phase II clinical trial for acute myelocytic leukemia, could inhibit TNBC cells and HER2-positive BC cells in distinct mechanisms (Figure 4) (Cuyàs et al., 2020; Wang et al., 2022). Compound **7** inhibited HER2-positive BC *via* reducing SOX2-driven breast cancer stem cells. In the mechanism, compound **7** disturbed the assembly between LSD1 and co-repressor RCOR1/CoREST *via* blocking the binding between LSD1 and FAD cofactor, and thus enhanced transcriptional repression of *SOX2* (Cuyàs et al., 2020). In TNBC, compound **7** suppressed the proliferation of TNBC cells *via* devitalizing androgen receptor (Wang et al., 2022).

### 4.2 Polyamine analogues

**FIGURE 5:**
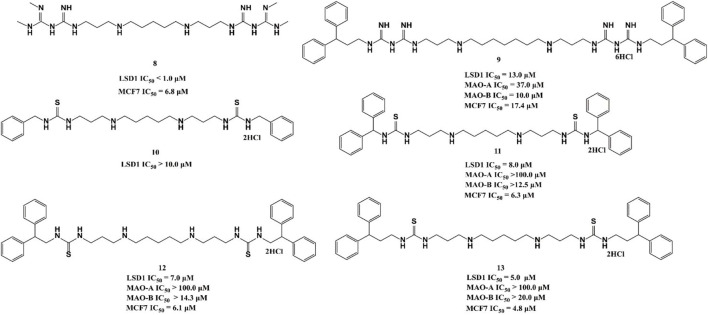
Chemical structures of polyamine analogue-based LSD1 inhibitors.

Polyamine analogues, previously identified as the FAD-dependent spermine oxidase inhibitors, were also found to inhibit LSD1 demethylase activity in 2007 (Huang et al., 2007). Bisguanidine **8** and biguanide **9** exhibited demethylase inhibitory activity by over 50% at 1 μM but both of them lack selectivity among MAOs (Nowotarski et al., 2015). Four (bis)-thioureidopropyldiamine compounds (**10**–**13**) showed improved selectivity compared with their lead compounds **8** and **9** (Figure 5). Among them**,** compound **13** exhibited the best selectivity and potency with IC_50_ values of 5.0 and 4.8 μM *in vitro*, respectively. In fact, **13** also showed much more potent anticancer activity against MCF7 cells than 2-PCPA (Nowotarski et al., 2015). Further study showed that compounds **10**–**13** suppressed the proliferation of MCF-7 cells *via* increasing H3K4me2 levels, which significantly upregulated tumor suppressor genes *p16*, *GATA4*, *HCAD*, and *SFRP2*. The docking assay indicated that **11** could form three hydrogen bonds with residues N535 and A539, and FAD within the LSD catalytic pocket. In addition, the hydrophobic interactions between hydrophobic residues in lining the LSD1 pocket and **11** also contributed to their binding ability.

### 4.3 Natural products

Natural products are one of the major sources in drug discovery due to their diverse chemical scaffolds and activity profiles (Fang et al., 2020; Yang et al., 2020; Yang et al., 2021a; Cheng et al., 2022b; Song et al., 2022). Many natural products and their derivatives have been found with *in vitro* inhibitory activity against LSD1.

**FIGURE 6:**
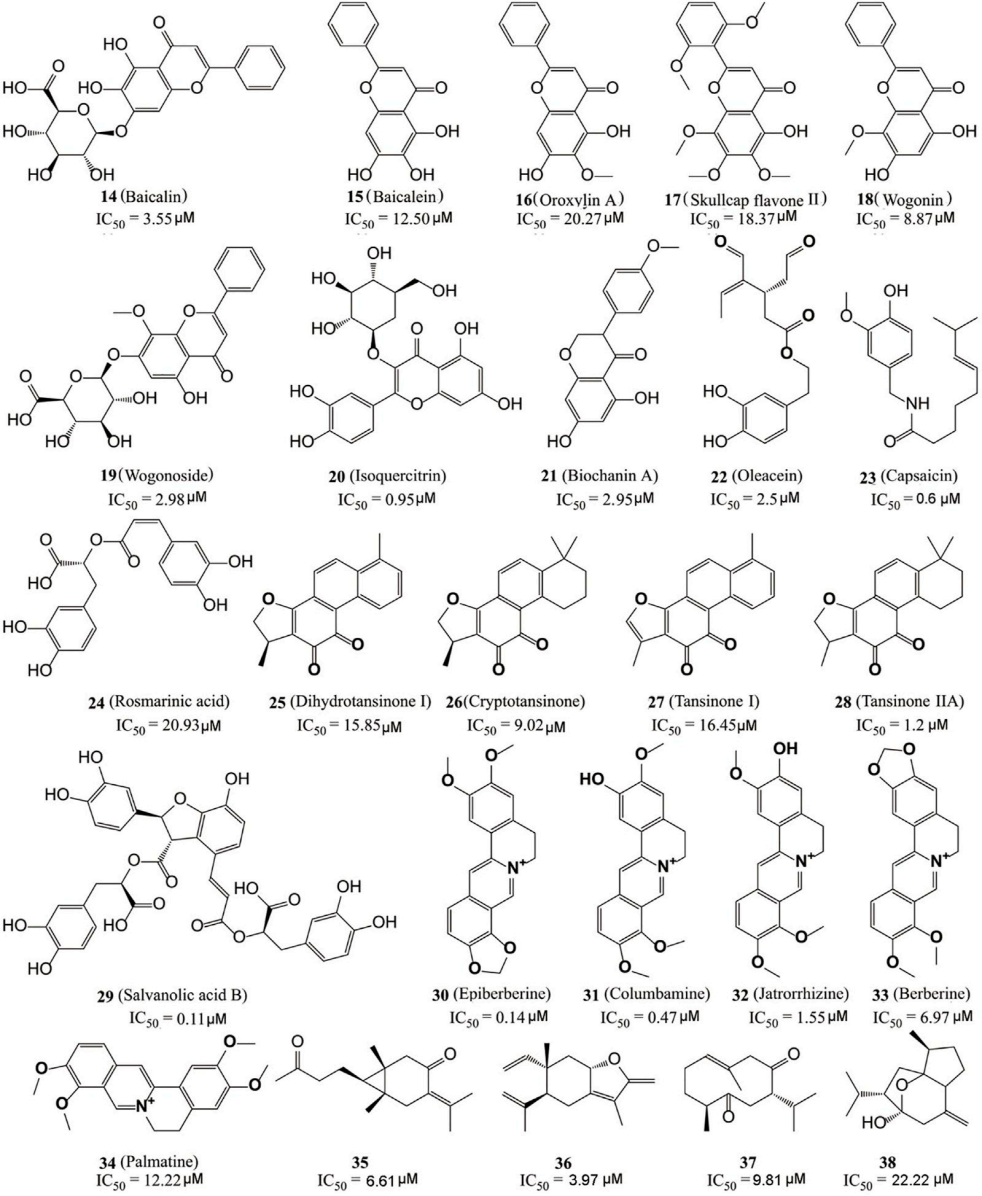
Structure of natural LSD1 inhibitors.

Kong’s group identified six flavonoid compounds (Figure 6, **14**–**19**) with inhibitory activity against LSD1 from *Scutellaria baicalensis* Georgi using countercurrent chromatography (CCC) (Han et al., 2018). Among them, compound **19** is the best LSD1 inhibitor with the *in vitro* IC_50_ of 2.98 µM and *in cellulo* IC_50_ of 17.94 µM against MDA-MB-231 cells. Isoquercitrin (Figure 6, **20**), a flavonoid compound with anti-BC activity extracted from *Bidens bipinnata L*, is an LSD1 inhibitor that inhibited proliferation of TNBC cell line MDA-MB-231 *via* activating mitochondrial-mediated apoptosis (Xu et al., 2019). Biochanin A (**21**), a dietary flavonoid from *Cicer arietinum* L, could inhibit the proliferation and metastasis of BC *in cellulo* and *in vivo* (Moon et al., 2008; Sehdev et al., 2009; Ren et al., 2018). Compound **21** was found to be effective and reversible with IC_50_ of 2.95 μM and it preferably suppressed LSD1 over MAO-A/B (>32 μM) (Wang et al., 2020). In gastric MGC-803 cells, Biochanin A induced the accumulation of H3K4me1/2 and inhibited cell growth moderately (IC_50_ = 6.77 µM) (Wang et al., 2020). Oleacein (Figure 5, **22**), a dihydroxy-phenol found in extra virgin olive oil, is a FAD competitive LSD1 inhibitor with IC_50_ of 2.5 μM *in vitro* (Cuyàs et al., 2019). Compound **22** reduced the stemness of BC stem cells *via* blocking the interaction between LSD1 and the methylated histone H3, disintegrating the assembled co-repressor complex LSD1/RCOR1/CoREST, disturbing the occupation of LSD1 to the *SOX2* promoter and finally reducing the *SOX2* level. Capsaicin (Figure 6, **23**), a bioactive compound from chili peppers with the broad spectrum of anticancer activity in various subtype of BC cells (Chou et al., 2009; Thoennissen et al., 2010; Wu et al., 2020; Chen et al., 2021), was found to act as reversible LSD1 inhibitor with an IC_50_ value of 0.6 μM (Jia et al., 2020). Compound **23** competitively occupied with FAD-binding sites within the catalytic pocket of LSD1, raised H3K4me1/2 levels and suppressed the proliferation and migration of BC cells.

The dried root of *Salvia miltiorrhiza* is a traditional Chinese medicine used for over 1,000 years to treat cardiovascular and cerebrovascular diseases, gynecological diseases, diabetes, and insomnia (Su et al., 2015; Jia et al., 2019; Shi et al., 2019). *S. miltiorrhiza* has also been reported to improve the survival rate of BC patients and several bioactive components (Figure 6, **24**–**29**) mediated its pharmacological actions *via* multiple anticancer pathways (Jin et al., 2021; Mahmoud et al., 2021). For example, rosmarinic acid (**24**) could suppress proliferation, metastasis and angiogenesis, and sensitize BC cells to paclitaxel *via* NF-κB-p53 pathways (Mahmoud et al., 2021). Dihydroisotanshinone I (**25**) inhibited the proliferation of BC cells *via* inducing their ferroptosis and apoptosis (Lin et al., 2019). Cryptotanshinone (**26**) inhibited migration through inactivating PKM2/β-Catenin signaling, and mediated drug resistance *via* reducing the oligomer formation of breast cancer resistance protein on the cell membrane, and thus blocking its efflux function (Zhou et al., 2020; Ni et al., 2021). Tanshinone I (**27**) inhibited the proliferation of MDA-MB-231 cells *via* activating the AMP-activated protein kinase mediating autophagic signaling (Zheng et al., 2020). Tanshinone IIA **(28**) sensitized BC cells to adriamycin *via* attenuates the stemness of BC cells by targeting the miR-125b/STARD13 signaling (Li et al., 2022). Recently, the six extracted monomeric compounds from roots of *S. miltiorrhiza* have been identified as LSD1 inhibitors with their IC_50_ values within the range 0.11–20.93 μM (Lin et al., 2020). Among them, salvianolic acid B (**29**) showed the best inhibitory activity against LSD1 (IC_50_ = 0.11 μM) and it demethylase-dependently inhibited the proliferation and migration of MDA-MB-231 cells with an IC_50_ value of 54.98 μM at 24 h against MDA-MB-231 cells.

*Coptis chinensis*, another traditional Chinese medicine widely used over 2,000 years for treatment of atherosclerosis, diabetes, and inflammation (Alami et al., 2020) has also been used to treat a varieties of cancers including BC (Wu et al., 2019), and isoquinoline alkaloids (Figure 6, **30**–**34**) have been identified as the main anticancer active components of *C. chinensis* with inhibitory activity against LSD1 (Yi et al., 2016). Epierberine (**30**) inhibited the proliferation and metastasis of BC cells *via* induced cell cycle arrest and induced apoptosis by regulating Wnt/β-catenin pathway (Dian et al., 2022). Jatrorrhizine (**32**) exhibited anti-proliferative activity *via* attenuating TNIK/Wnt/β-catenin signaling in BC cells with IC_50_ values of 11.08, 17.11, and 22.14 μM against MCF-7, MDA-MB-231, and 4T1 cells, respectively (Sun et al., 2019). Berberine (**27**) sensitized the BC cells to chemotherapeutic agents *via* reducing XRCC1-mediated base excision repair (Gao et al., 2019). Palmatine (**34**) reduced the lung metastasis of TNBC *via* downregulating metastasis-associated protein 1 (MTA1) and increasing p53 level (Ativui et al., 2022). Recently, Li Z. R. et al. (2020) found that five protoberberine alkaloids (**30**–**34**) also showed the inhibitory activities against LSD1. All the IC_50_ values of them were as low as micromoles and highly selective to LSD1 over MAOs.

Recently, Ren et al. (2021) also identified four sesquiterpene-based LSD1 inhibitors (compounds **35**–**38**) with their IC_50_ values within the range 3.97–22.22 μM from zedoary turmeric oil using CCC strategy. Compound **36** had the best inhibitory activity (IC_50_ = 3.95 μM) of them and also exhibited the anti-metastasis activity against MDA-MB-231 cells.

### 4.4 Propargylamine derivatives

**FIGURE 7:**
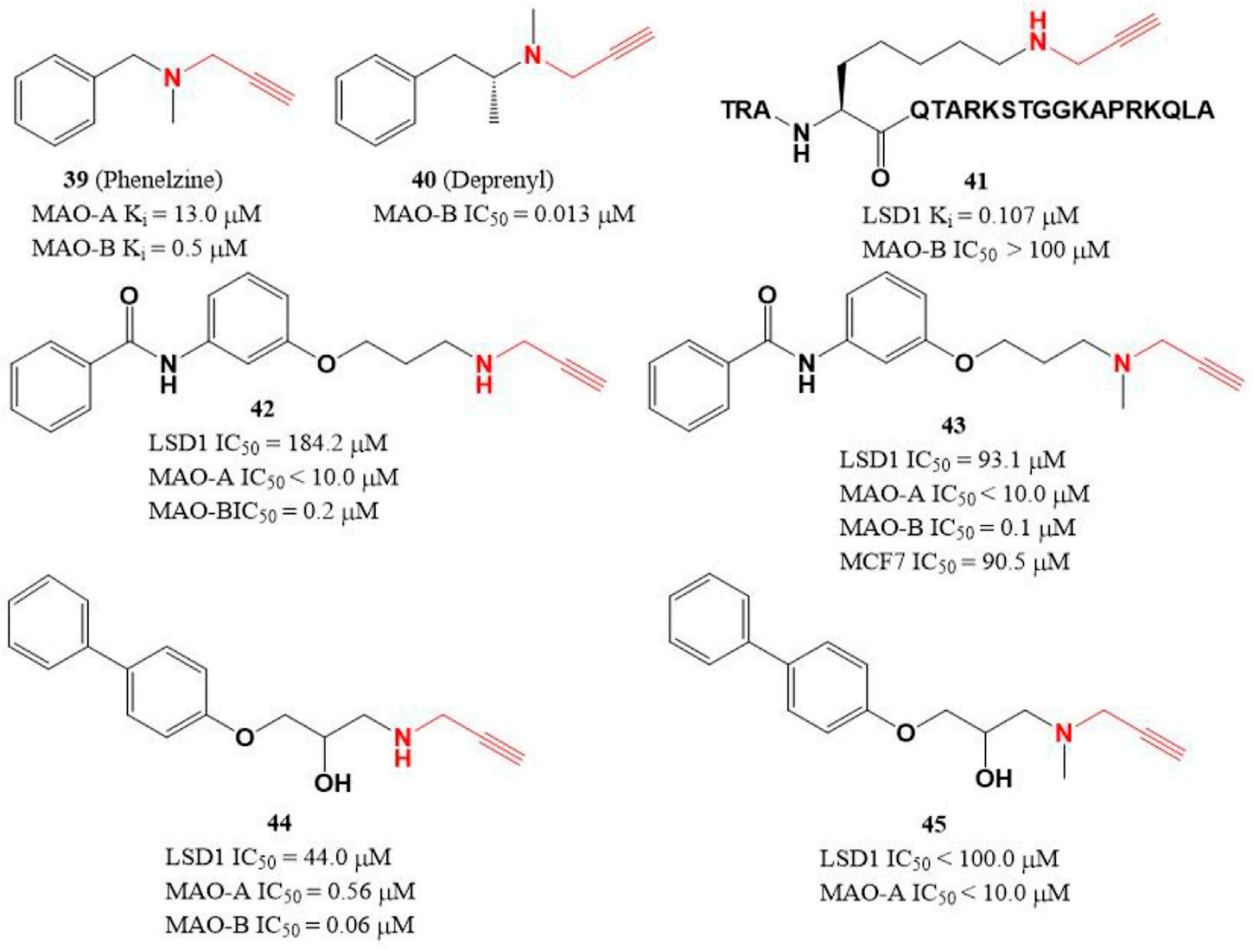
Structure of propargylamine-based LSD1 inhibitors.

Due to the similar structure between MAOs and LSD1, two MAOs inhibitors (Figure 7, **39** and **40**) with active propargylamine group also showed a weak inhibitory activity against LSD1 in the millimolar range (Lee et al., 2006; Matos et al., 2009). To improve the potency of LSD1 inhibitor, a covalent and irreversible LSD1 inhibitor (**41**) was designed through combining the active propargylamine group with N-terminal 21 amino acids of LSD1 substrate H3, compound **41** selectively inhibited LSD1 demethylase activity with a Ki value of 0.107 μM (Culhane et al., 2006). Based on the structure of **41**, Schmitt et al. (2013) designed and synthesized a set of with propargyl warhead (Figure 7, **42**–**45**). Among them, compound **44** was the best LSD1 irreversible inhibitor with IC_50_ of 44.0 μM *in vitro*, while **43** has the best anti-proliferative activity against MCF7 cells (IC_50_ = 91.5 μM) for 24 h (Schmitt et al., 2013). The docking analysis of the binding mode between **42** and LSD1 showed that residues Y761 and D555 formed hydrogen bonds with the amine of N-propargylamine warhead and the amide nitrogen atom of the benzamide moiety, respectively. The aromatic substituents extensively form hydrophobic and T-shaped aromatic interactions with F558, F560, Y807, and H812.

### 4.5 Benzohydrazide derivatives

**FIGURE 8:**
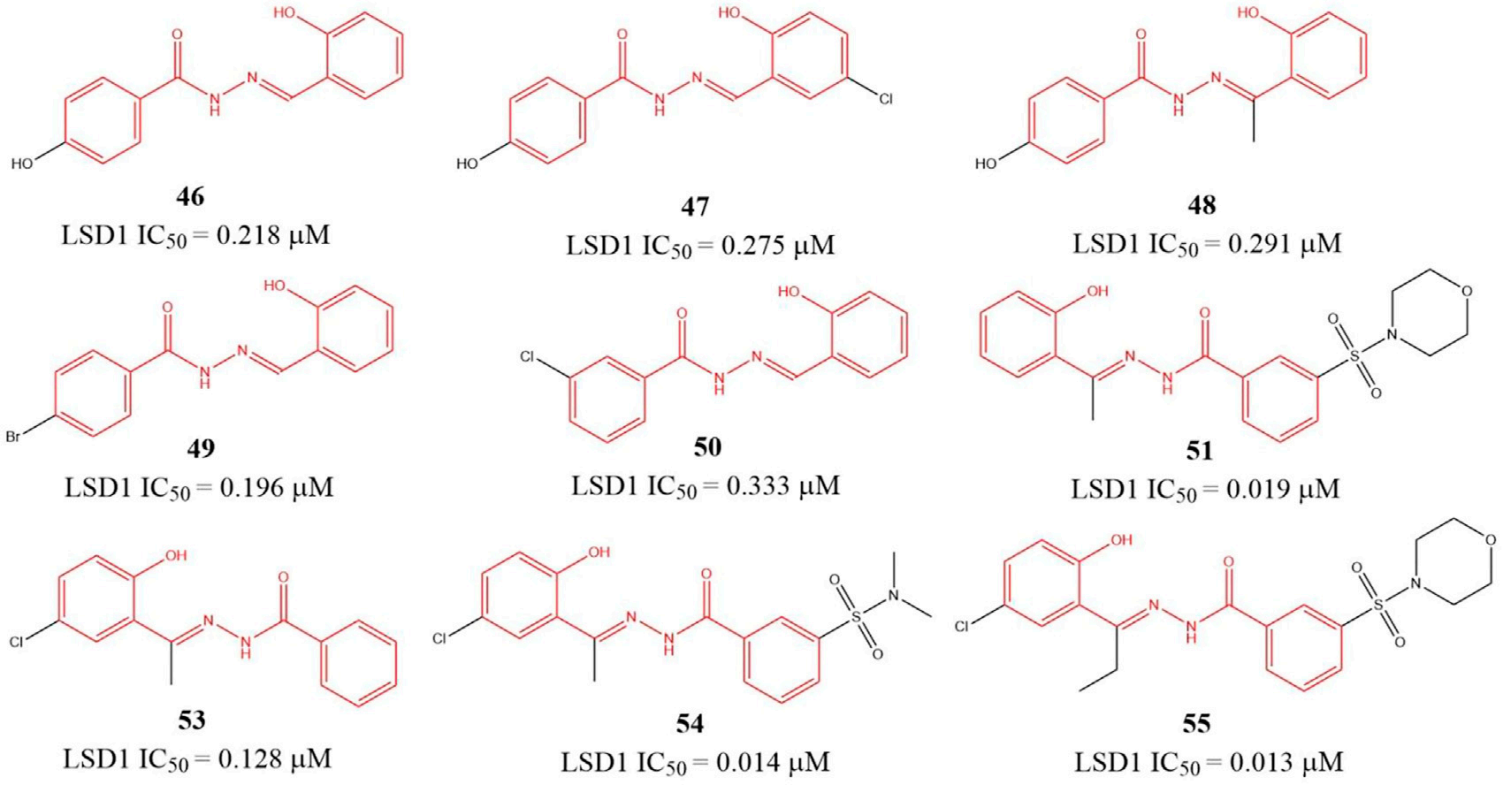
Structure of benzohydrazide-based LSD1 inhibitors.

Sorna et al. (2013) identified six benzohydrazides (Figure 8, **46–51**) IC_50_ values within 0.19–0.333 μM range based on virtual screening from a compound library containing 2,000,000 compounds and biochemical assay. Then, three compounds **53–55**, with IC_50_ of 0.128, 0.013, and 0.014 μM, respectively, were gotten though structure-based optimization. Compound **55**, the best potent LSD1 inhibitor of them, is reversible and specific for LSD1 and inhibits the proliferation and survival of seven BC cell lines with IC_50_ from 0.468 to 2.730 μM range, which is more potent than a known MAO inhibitor 2-PCPA. Further study showed that **55** suppressed the proliferation of BC cells *via* reducing Sox2 expression, promoting G1 cell cycle arrest, and inducing the expression of differentiation-related genes in demethylase-dependent manner (Zhang et al., 2013). The binding mode analysis using ICM software showed that **55** could form three H-bonding interactions with the residues G314, R350, and Y510 *via* its benzohydrazide scaffold.

### 4.6 Phenyl oxazole derivatives

**FIGURE 9:**
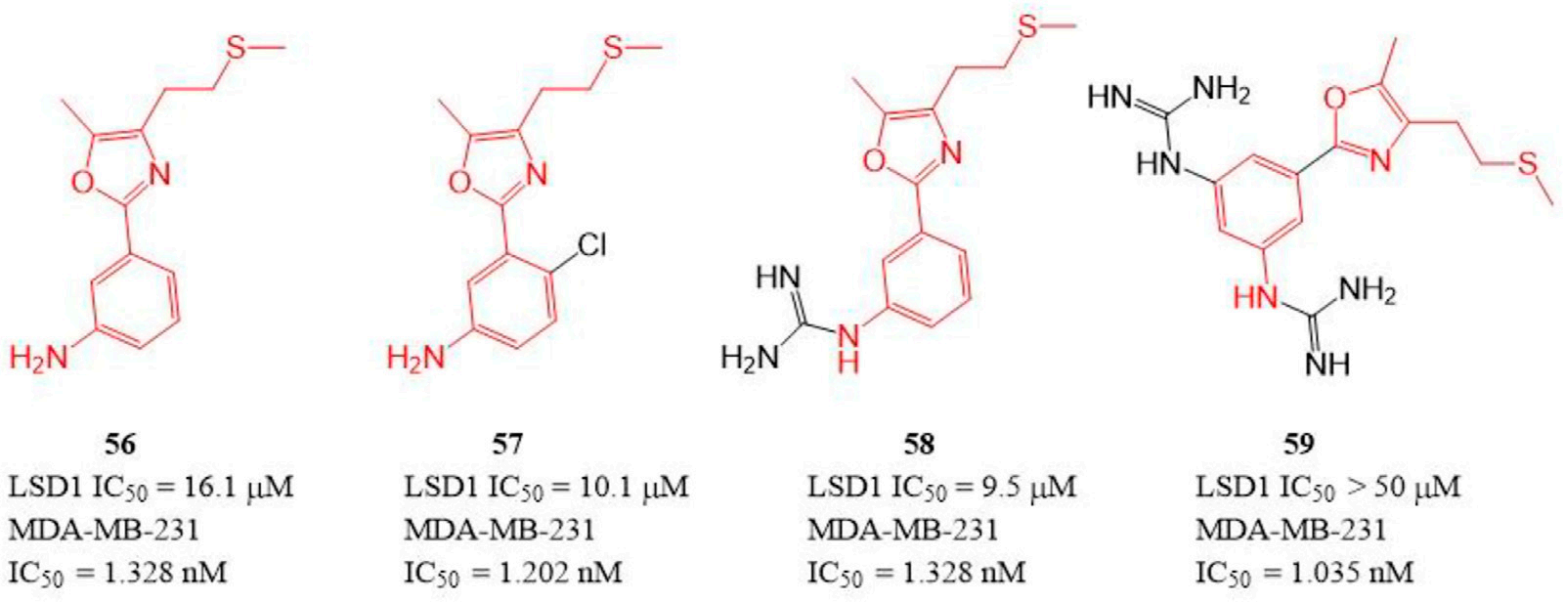
Structure of phenyl oxazole-based LSD1 inhibitors.

The polyamine/guanidine and methionine were often key pharmacophores for designing MAOs inhibitors. Dulla et al. designed a series of compounds (Figure 9, **56**–**59**) with related pharmacophores using oxazole as a linker (Dulla et al., 2013). The results showed that compounds **56**–**58** exhibited good *in vitro* inhibitory activity against LSD1 with IC_50_ of 16.1, 10.1, and 9.5 μM, respectively. Interestingly, all the synthesized compounds including compound **59** showed an excellent cytotoxicity activity against MDA-MB-231 cells (IC_50_ = 1.035–1.328 nM) *in cellulo,* which suggested these compounds have other targets contributing their anticancer activity. Docking analysis showed that the free -NH2 and the oxazole nitrogen of compound **56** formed H-bonding with residues T624 and R316 of LSD1, respectively, and its oxazole ring is involved in a π-cation interaction with residue R316. In case of **57,** its free -NH2 formed a H-bond with S760, the sulfur atom formed H-bond with M332 and V333, and the phenyl ring interacted with W751 and Y761 residues *via* π-π stacking. In case of **57**, its replaced guanidine group could form two H-bonds with residues E308 and R310 whereas its phenyl ring interacted with R316 *via* π-cation interaction.

### 4.7 Dual-target inhibitors and combined therapy

**FIGURE 10:**
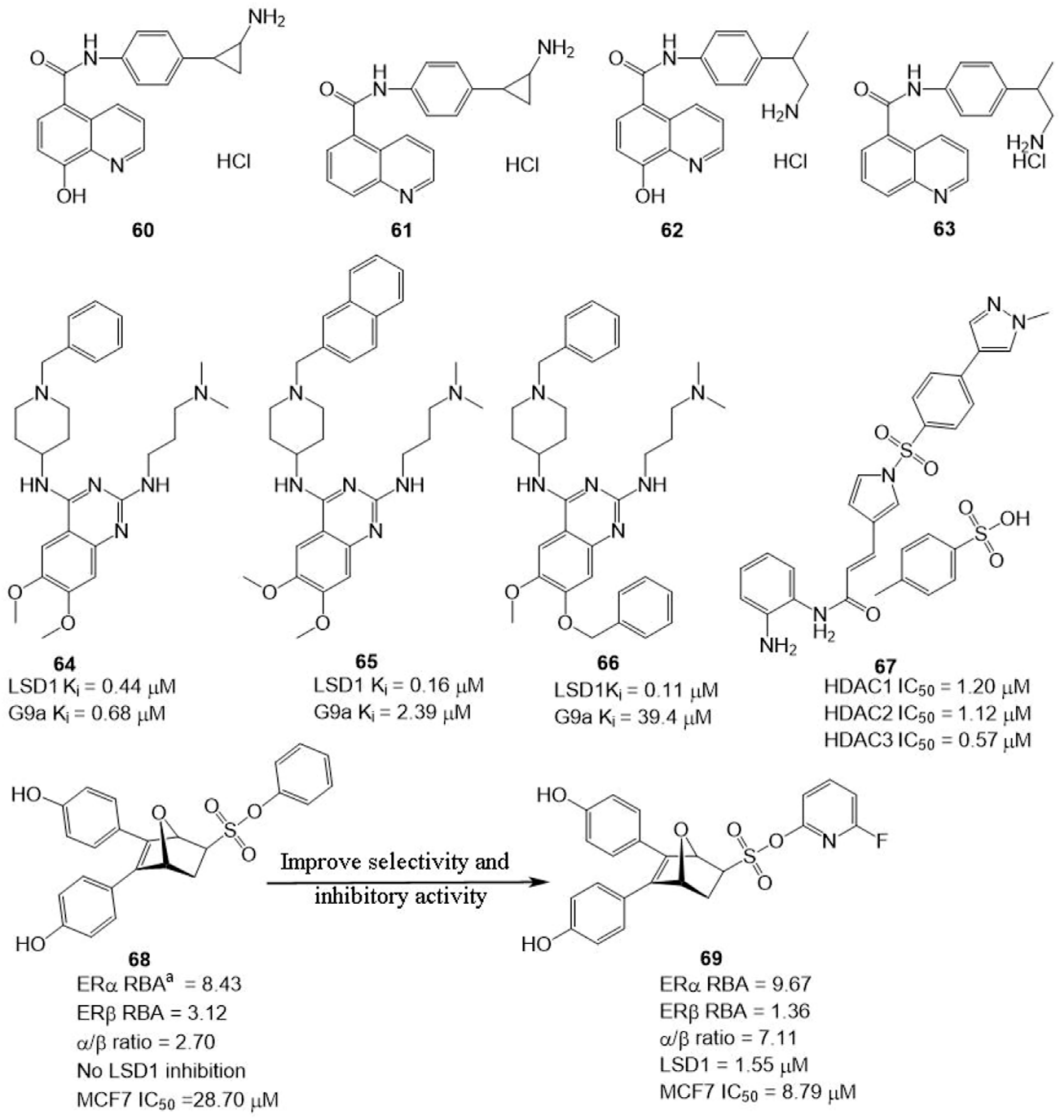
Structure of reported dual-target inhibitors. ^a^ RBA values = ${IC}_{50}^{E2}$ / ${IC}_{50}^{Compound}$ × 100 ± the range (RBA value of E2 as 100%).

In BC, LSD1 and KDM6A have been found to co-express and co-localize with ER, and regulate hormone receptor signaling (Benedetti et al., 2019), suggesting that developing dual-targeting agents against the two proteins are potent strategy to improve the potency of LSD1 inhibitors. Based on this hypothesis, Altucci’s group designed four dual-KDM inhibitors (Figure 10, **60**–**63**) targeting LSD1 and KDM6A. Among them, compound **61** exhibited the best anti-BC activity with no significant toxicity and good oral potency. In the mechanism, **61** induced cell arrest and apoptosis of hormone-positive BC *in cellulo* and *in vivo* and exhibited lower toxicity against non-cancerous cells (HaCaT) compared with clinical HDAC inhibitor Vorinostat. Additionally, it also reduced the resistance to endocrine therapies, suggesting dual-target is a feasible and potent strategy to overcome drug resistance for BC therapy.

Compound **64** was identified as a non-covalent dual LSD1/G9a inhibitor with anti-leukemia activity (Speranzini et al., 2016; Menna et al., 2022). Mai’s group optimized the structure of **64** *via* modifying its quinazoline core and got two non-covalent, and more potent LSD1/G9a inhibitors (Figure 10, **65** and **66**). Compared to lead compound **63**, compounds **65** and **66** exhibited a better inhibitory activity against LSD1 but reduced the inhibitory activity against G9a. Further study showed that **65** and **66** showed better anticancer activity against MDA-MBA-231, THP-1, and MV4-11 cells without significant toxicity to non-cancer AHH-1 cells compared with epigenetic drug UNC0638, which suggested that the LSD1 antagonistic activity of LSD1/G9a inhibitors endorsed with anticancer activity (Menna et al., 2022).

LSD1 has been found to promote BC proliferation *via* interacting with histone deacetylases (HDACs) (Cao et al., 2017). Huang’s group found that HDAC inhibitor sulforaphane suppressed the proliferation of BC cells *via* blocking activity of upstream transcription factor 1 (USF1) and promoting ubiquitination degradation of LSD1, and LSD1 inhibitor significantly sensitized sulforaphane to BC *in cellulo* and *in vivo* (Cao et al., 2018), which inhibited that the combined therapy or discovery of LSD1/HDAC5 inhibitors is a potential strategy for BC treatment. Zylla et al. (2022) study found that HDAC/LSD1 inhibitor **67** (Figure 10) exhibited anti-proliferative and anti-metastatic activity against TNBC *in cellulo* and *in vivo*.

Currently, hormonal drugs and chemotherapy have been widely combined in the utilization for BC therapy in clinic. But combined strategies also showed undeniable disadvantages during clinic use. To overcome this phenomenon, Zhou’s group developed a set of dual-target inhibitors based compound **68**. Among these conjugators, compound **69** has the best *in vitro* and *in cellulo* activity with IC_50_ values of 9.67, 1.36, 1.55, and 8.79 μM against ERα, ERβ, LSD1, and MCF7, respectively (He et al., 2020). Most notably, **69** exhibited better anti-BC activity in MCF-7 cells than clinical agent 4-hydroxytamoxifen.

## 5 Discussion and future prospects

Mounting evidence supports that LSD1 is overexpressed in many subtypes of BC and promotes their proliferation (Pollock et al., 2012; Yang et al., 2018d; Xu et al., 2019; Wang et al., 2022), differentiation (Wu et al., 2013; Zhang et al., 2013; Ji et al., 2021), metastasis (Li et al., 2011; Qiu et al., 2018; Zheng et al., 2018; Hu et al., 2019; Gong et al., 2021), and drug resistance (Bennani-Baiti, 2012; Verigos et al., 2019; Zhou et al., 2021a; Liu et al., 2022), which makes LSD1 become a promising target for BC therapy. But the detailed mechanisms of the LSD1 in BC progression are unclear and more potential anti-tumor pathways or downstream genes are yet to clarify due to the heterogeneity of varieties of BC subtypes. For example, metastasis and drug resistance are two main factors responsible for BC-caused death in clinic (Cheng et al., 2021; Cheng et al., 2022a). Although there are several reported researches on the roles of LSD1 in BC metastasis and drug resistance, the specific functions of LSD1 in these two cancer cell events are yet to be investigated. In addition, many studies showed that LSD1 functioned as an oncogene or suppressor gene in BC progression dependent on its transcriptional regulatory activity *via* assembling into different complexes with its client proteins. Therefore, mapping the protein-protein interactome of LSD1 is a potential strategy to further clarify its function in BC development (Yatim et al., 2012; Yang et al., 2022). Meanwhile, the exploration on the functions of LSD1 in homeostasis is also imperative to avoid the potential health risks during the development and advancement of clinical trials of LSD1 inhibitors. Moreover, most of the current studies about LSD1 functions mainly focus on its histone demethylase activity, and few works are available about the roles of non-histone substrates, epigenetic modifications, and non-enzyme activity of LSD1 in BC progression (Feng et al., 2016; Liu et al., 2020a; Gong et al., 2021). Thus, it is imperative to further carry out research in these areas.

Currently, some LSD1 inhibitors have entered clinical trials to combat small lung cancer cells and acute myeloid leukemia and several of them showed encouraging results (Fang et al., 2019). But there is no LSD1 inhibitor in clinical trials for BC. Many factors contribute to this phenomenon apart from the complex etiological factor of BC. First, most of the reported LSD1 inhibitors have poor selectivity, which adds to the uncertainty of drug therapy. Second, some LSD1 inhibitors such as compounds **56**–**59** exhibited intracellular activity inconsistent with their inhibitory activity against demethylase activity (Dulla et al., 2013), which suggested that there are potential off-target effects and unpredictable risks of some identified LSD1 inhibitors, when they were advanced into clinical trials for BC therapy. Third, like other enzyme inhibitors (Yang et al., 2018c; Wu et al., 2018; Yang et al., 2019; Yang et al., 2021c; Yang et al., 2021d), most of the reported LSD1 inhibitors were only detected in anti-BC activity *in vitro* or *in cellulo* assays with a dearth of the studies about *in vivo* toxicology, pharmacokinetics, and effectiveness in animals. Finally, due to the existence of several alternative signaling and isoenzymes in BC cells, LSD1 inhibitor used alone may be not sufficient to achieve the desired therapeutic effect sometimes. To solve these dilemmas, pharmaceutical chemists and pharmacologists have proposed several strategies. Given allosteric regulation is a common characteristic for metabolic enzymes (Kremer and Lyssiotis, 2022), identifying allosteric pockets or sites and developing corresponding inhibitors is a feasible strategy for the discovery of LSD1 inhibitors. Targeting the protein–protein interaction (PPI) is also an effective method to improve the selectivity of enzymes (Yang et al., 2018a; Cheng et al., 2020; Yang et al., 2021a; Yang et al., 2021c; Yang et al., 2021d; Yang et al., 2022), blocking the interaction between LSD1 and its client proteins may be also a useful strategy to develop selective LSD1 inhibitors. Metal complexes have showed promising *in vivo* activity for BC treatment (Yang et al., 2018b; Cheng et al., 2022b), and Yang et al. (2017) have also identified a rhodium-based LSD1 inhibitor with *in cellulo* anticancer activity against prostate cancer, which suggested this kind of compound is a unique source for the discovery of potent and selective LSD1 inhibitors against BC treatment. Several drug design strategy such as computer aided drug optimization and proteolysis targeting chimera (PROTAC)-strategy have been introduced to discover lead compounds against LSD1 with better biocompatibility and *in vivo* potency (Liu et al., 2020b; Martín-Acosta and Xiao, 2021). Moreover, combined therapy and developing dual-target inhibitors have also been used to improve the anticancer potency and overcome drug resistance of LSD1 inhibitors (Ota et al., 2016; Cao et al., 2018; Benedetti et al., 2019; Qin et al., 2019; Verigos et al., 2019; Liu et al., 2022). Drug delivery by nanocarriers is a potent strategy to increase drug utilization rate, improve potency, and reduce toxicity and drug resistance of single agent or combined therapy (Singh and Mitragotri, 2020; Vallet-Regí et al., 2022). LSD1 has been found to exhibit its activity by promoting gastric cancer cell stemness *via* delivered by endogenous small extracellular vesicles *in vivo* (Zhao et al., 2021), and the drug delivery of LSD1 siRNA using nanocarriers has exhibited potent anticancer activities in several studies (Suzuki, 2019; Li B. et al., 2020; Jiang et al., 2021).

In a word, LSD1 plays a crucial role in BC progression and drug resistance, and its inhibition is a potential anti-BC strategy due to its efficacy in current preclinical studies. Therefore, the discovery of more specific LSD1 inhibitors is imperative to deepen our understanding of the role of LSD1 in BC tumorigenesis and verify the feasibility of targeting LSD1 as an anti-BC therapy in clinic.
